# Overtreatment of cruciate ligament injuries

**DOI:** 10.3109/17453674.2010.525192

**Published:** 2010-10-08

**Authors:** Per Aspenberg

Once an unvalidated surgical procedure has become common, widely known in the general population, and popular among “celebrities”, it is regarded as too late to evaluate its efficacy. Patients will be unwilling to risk control treatment, and doctors will be unwilling to recruit. To challenge such procedures is truly heroic. Orthopedics has seen no less than 3 such heroic challenges only in the last few years, all of which have led to publications in New England Journal of Medicine and which have caused a healthy amount of turmoil within our specialty. First came the randomized trial of arthroscopic debridement versus sham surgery for osteoarthritis of the knee (Moseley 2002), with its followers (Herrlin 2007, Kirkley 2008), then the two trials of vertebroplasty versus sham surgery for vertebral compression fracture (Buchbinder 2009, Kallmes 2009). Both procedures appeared to produce nothing but placebo effects. The third challenge addresses early cruciate ligament reconstruction after injury in young active individuals (Frobell 2010). A structured training program was used, with comparison of early or optional delayed reconstruction. After two years, 61% of the patients in the latter group were spared the risks and discomfort of surgery, but had an identical self-reported function. The remaining 31% underwent delayed reconstruction. There was no difference in any relevant clinical variable, either between the two initial groups or between early and delayed reconstruction. Clinically meaningful differences at two years could be excluded.

This study is the fruit of decades of work by Stefan Lohmander's group, who has focused on long-term studies on the development of knee osteoarthritis since the 1980s. Their persevering questioning of “clinical truths” in the field had slowly spread to the colleagues and sports physicians in their region, so that it became possible to recruit even athletes for a randomized trial. In fact, there were more patients who refused to participate because they wanted to avoid surgery than because they wanted a reconstruction to be performed. Thereafter, the authors worked for almost 10 years to conduct the study, which meets the highest requirements for scientific rigor. Read and learn! More data have been collected on these patients, suggesting a possible detrimental effect of reconstruction (Frobell 2009), and longer follow-up will probably yield important information.

Of course, there has been criticism—mainly targeting the generalizability of the results (Levy 2010). It is possible that the results would have been different if only subjects with a certain (narrowly defined) activity level or type had been included. However, most of this criticism misses the point: randomized trials produce scientific data for interpretation, not treatment algorithms. Results are never completely generalizable. The conclusion of Frobell's results is that most patients who are operated on early after anterior cruciate ligament injury undergo the procedure in vain. This information can be used to justify different treatment algorithms. Some would let all patients undergo a structured rehabilitation program before eventually recommending surgery for those deemed to need it, whereas others would suggest that a minority of cases, defined by a certain activity level, age or personality, should be recommended early reconstruction. Nothing can replace clinical judgement, but judgement without data is worthless. The value of Frobell's study is that it provides a foundation for better clinical decisions. And as most patients—even in this young and active group—appear to undergo early reconstruction in vain, it is clear that starting with a structured training program is the treatment of choice for the vast majority.
